# Development of Venous Thromboembolism After COVID-19 mRNA-1273 Vaccine Inoculation

**DOI:** 10.7759/cureus.22179

**Published:** 2022-02-13

**Authors:** Abdullahi E Mahgoub, Dominic Awuah, MurtazaShabbir Hussain, Smit Deliwala, Ghassan Bachuwa, Mariam Younas

**Affiliations:** 1 Internal Medicine, Hurley Medical Center, Flint, USA; 2 College of Human Medicine, Michigan State University, Lansing, USA; 3 Infectious Diseases, Hurley Medical Center, Flint, USA

**Keywords:** covid vaccines, vaccine induced thrombosis, venous thromboembolism, mrna-1273 vaccine, covid-19

## Abstract

A 79-year-old man suddenly developed right lower extremity (RLE) pain and swelling a few days after receiving his 1st dose of the mRNA-1273 COVID-19 vaccine. Despite this, he proceeded to receive the 2nd dose of his mRNA-1273 COVID-19 vaccine. Investigations confirmed extensive acute deep venous thrombosis and a concurrent acute pulmonary embolism. Therapeutic anticoagulation was initiated and he was eventually discharged home on supplemental oxygen. The overall benefits of the vaccine in curbing severe disease overwhelmingly outweigh the handful of cases of reported adverse events. To our knowledge, this is one of the first few cases of provoked venous thrombosis after receiving the mRNA-1273 COVID-19 vaccine during the pharmacovigilance period.

## Introduction

The coronavirus disease 2019 (COVID-19) has reached catastrophic levels - over 359 million global cases and 5.6 million deaths by the end of January 2022 [1], with a mortality rate nearing 11%, higher than historical pandemics [2]. Although as time goes, we are seeing new modalities of treatment, still prevention strategies remain crucial in the fight against the disease. The human response to the pandemic has been robust, with over nine billion vaccine doses administered successfully within one year period [1]. This global reach was possible due to the novel development of messenger RNA (mRNA) vaccines, (BNT162b2 and mRNA-1273). Although the incidence of adverse events is exceedingly rare, reporting during the pharmacovigilance period can be utilized to collect more extensive data to assess the effect of these vaccines [3,4]. We hypothesize that the mRNA-1273 vaccine led to the development of a systemic reaction in the form of venous thrombosis in a patient with otherwise no risk factors for venous thromboembolism (VTE).

## Case presentation

**Figure 1:**
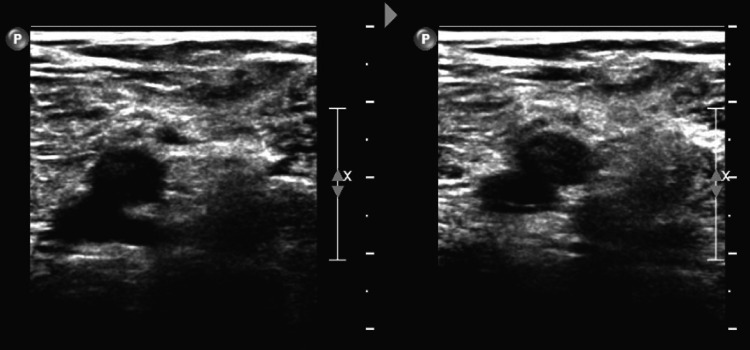
Doppler venous ultrasound of the right lower extremity with one of the trunks not compressible demonstrating an acute deep vein thrombosis in the right trunks.

**Figure 2:**
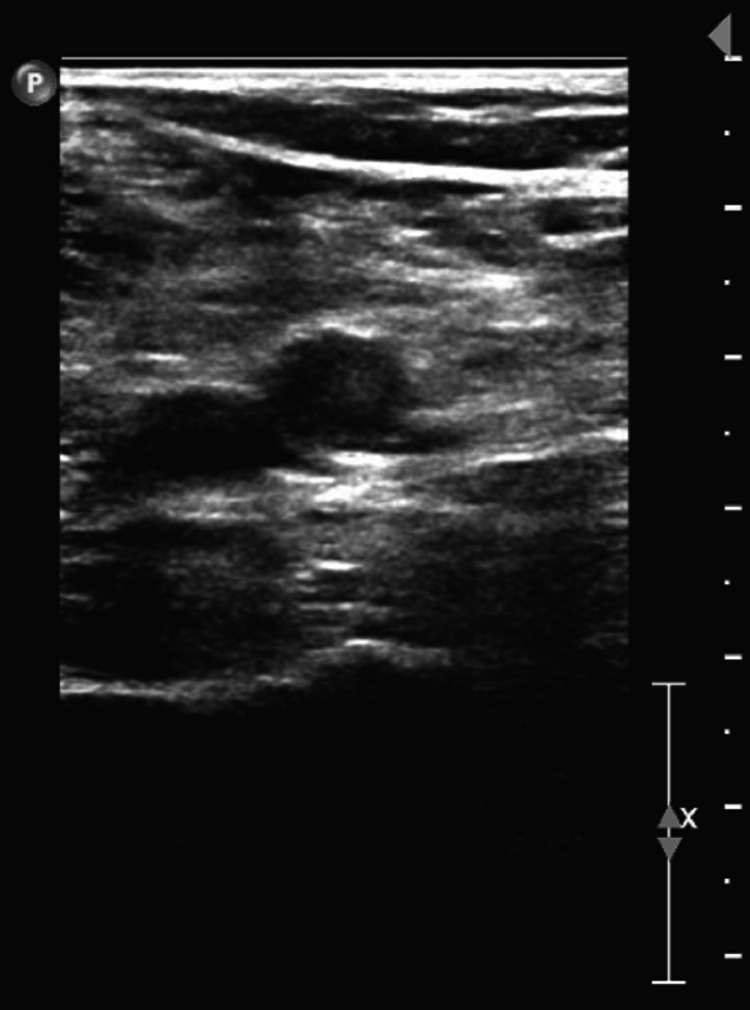
Doppler venous ultrasound of the right lower extremity with the right popliteal vein not compressible demonstrating an acute deep vein thrombosis in the right popliteal vein.

**Figure 3:**
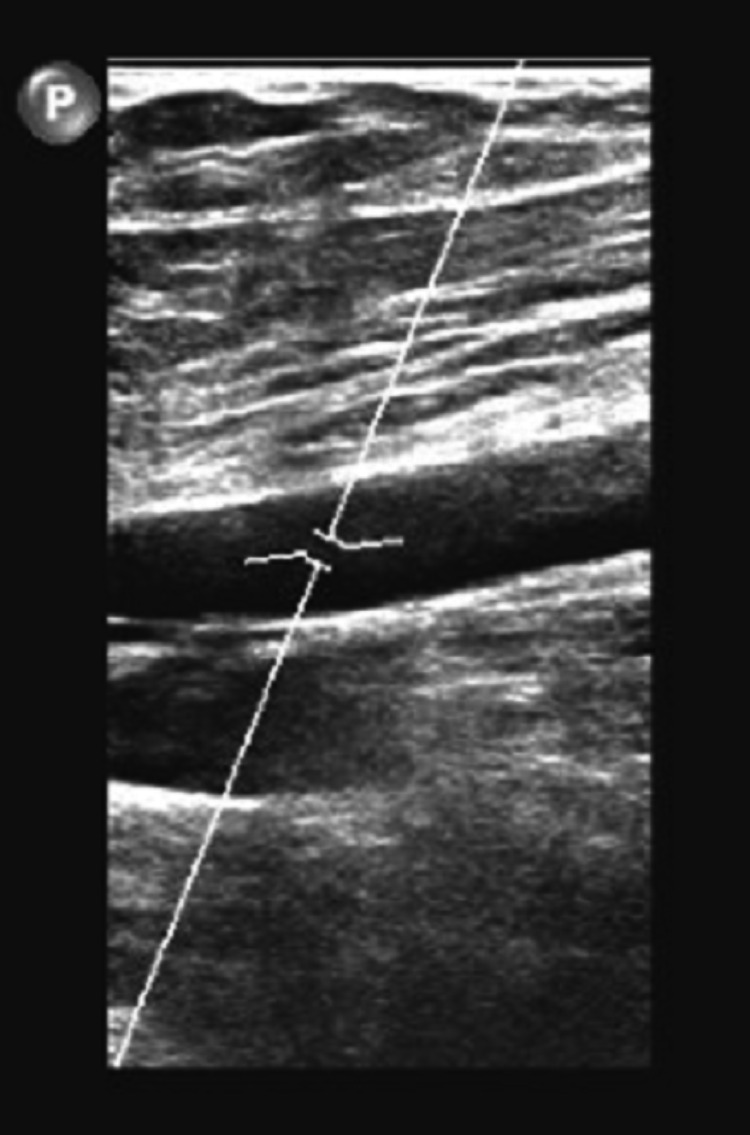
Doppler venous study of the right lower extremity with the right femoral vein not compressible demonstrating an acute deep vein thrombosis in the right femoral vein.

**Figure 4:**
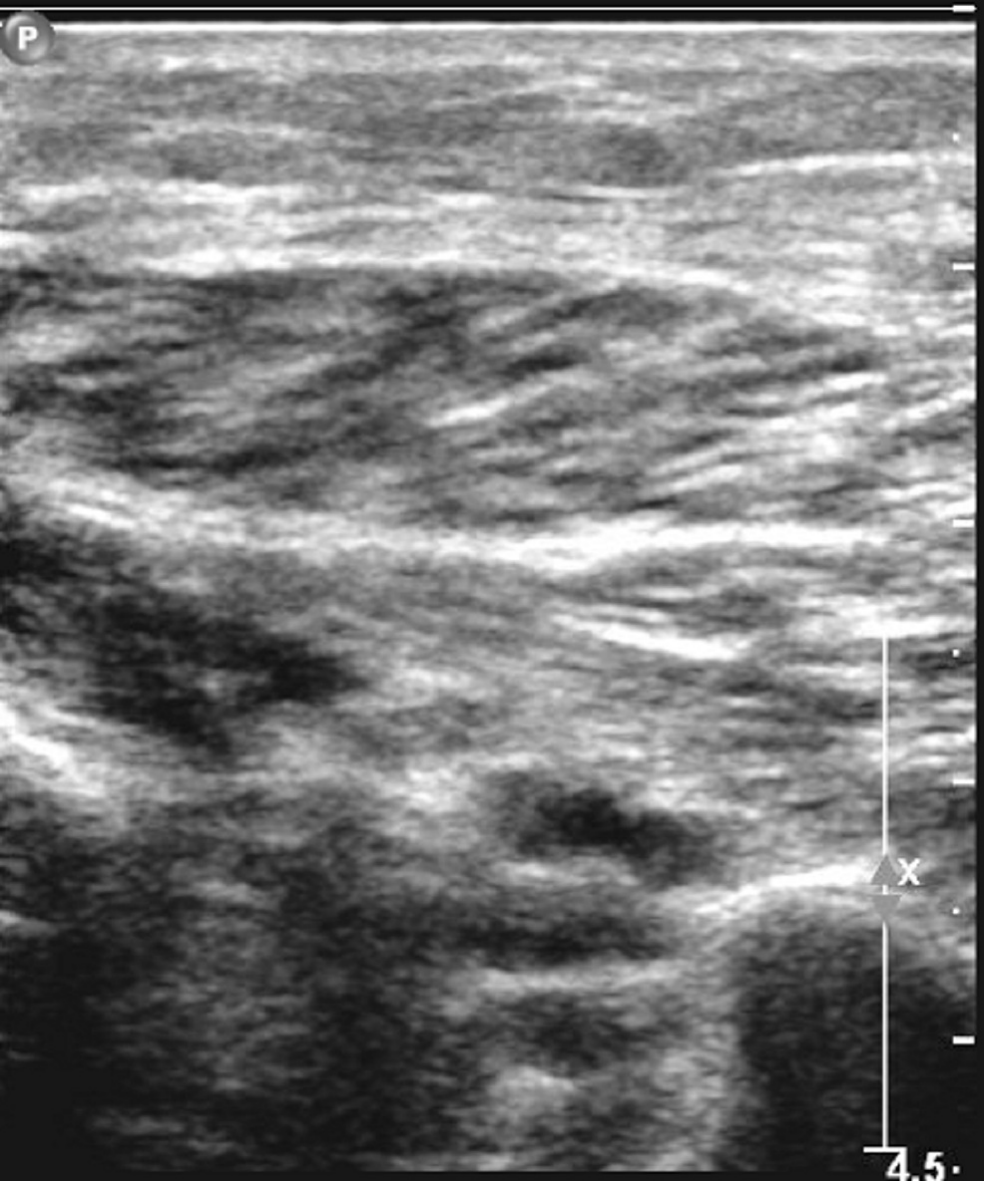
Doppler venous study of the right lower extremity with the right peroneal vein not compressible demonstrating an acute deep vein thrombosis in the right peroneal vein.

A 79-year-old male with an unremarkable past medical history and completed lifetime health screenings had his first dose of the mRNA-1273 COVID-19 vaccine on May 12, 2021. Three days later, he developed some pain with swelling in his right lower extremity (RLE) below the knee. He was seen by his primary care physician who ordered a duplex venous ultrasound of the lower extremities; however, it was delayed because of scheduling problems. Meanwhile, he proceeded to receive his 2nd dose of the mRNA-1273 COVID vaccine 28 days after the 1st vaccine dose on June 9, 2021. Worsening of his RLE pain and increased shortness of breath led him to complete the venous doppler ultrasound on June 10, 2021, where an extensive DVT was found along the RLE (Figures 1-4).

The patient subsequently presented to our emergency department (ED). His first set of vital signs upon arrival recorded as blood pressure of 187/83 with a heart rate (HR) of 85 beats per minute, he was afebrile but hypoxic to 87% on room air requiring supplemental oxygen. He was tested for COVID-19 infection with reverse transcription-polymerase chain reaction (RT-PCR) which was negative. Shortly after presentation, he developed atrial fibrillation with rapid response rate, HR reaching 142 before being controlled with beta-blockers. His right leg below the knee was erythematous, warm, and swollen with mild tenderness and intact distal pulse. He denied using tobacco products, alcohol, or illicit substances. He works as a construction worker occasionally and maintains an active lifestyle. He denied history of recent trauma, surgery or prolonged hospitalization, uninterrupted long-distance traveling, or a personal or family history of blood clots.

An emergent computed tomography angiography (CTA) of the chest revealed an acute subsegmental right lower lobe pulmonary embolism. Echocardiogram revealed normal ejection fraction without right ventricular strain or structural abnormalities. Troponin was normal at 0.004 ng/ml. His hematologic, metabolic, electrolyte, and coagulation panels were within standard limits except for D-dimer which was elevated to 1.22 ug/ml (normal 0.00 - 0.49 ug/ml). 

He was immediately anticoagulated with intravenous heparin and admitted to a closely monitored unit. Due to persisting hypoxia, he required supplemental oxygen on discharge. Apixaban was chosen for ambulatory anticoagulation, initial dose was 10 mg twice daily for two weeks to be followed by 5 mg twice daily for three months and was advised to follow-up with his primary care physician and pulmonologist.

## Discussion

mRNA vaccines are revolutionary gene-based vaccines that instruct the host cells to express spike proteins that closely mimic the natural COVID-19 proteins, triggering an immune response and development of immunity. In December 2020, BNT162b2 and mRNA-1273 were granted emergency use authorization by the US Food and Drug Administration. The reactogenicity of these vaccines, such as fatigue, headache, myalgias, are more common, while their systemic reactions are exceedingly rare, including anaphylaxis [4]. We report a case of venous thrombosis temporally associated with the administration of the mRNA-1273 COVID-19 vaccine in a patient with otherwise no known risk factors for development of DVT. 

In a recent post-vaccination database, there were 213 cases of cerebral venous thrombosis - 88% were attributed to ChAdOx1, 12% to BNT162b2, and one case by mRNA-1273 [5]. Another study found the incidence of thromboembolic events of 1 per 222,951 vaccinated in a cohort of 13.6 million women aged <50 years [6]. In the assessment report by the European Medicines Agency, two cases of DVT were reported in the vaccine group compared to none in the placebo group [7]. These numbers are reassuring during the pharmacovigilance period.

Scientists are widely discussing vaccine-induced thrombotic thrombocytopenia (VITT) and its role in the multiple incidents of thrombosis following COVID-19 vaccination [8]. In addition to thrombocytopenia, VITT is usually characterized by the presence of a positive anti-platelet factor 4 (PF4) IgG antibodies, which mimic the antibodies found in heparin induced thrombocytopenia (HIT). VITT usually follows the administration of the adenoviral vector-driven vaccines like Ad26.COV2.S and ChAdOx1 [9]. Although some cases demonstrate that VITT can happen after receiving mRNA-1273 vaccine [10,11]. Also, researchers were able to establish a link between the development of DVT and another mRNA COVID vaccine, BNT162b2 [12]. 

Although it may be argued that in our patient PF4 antibodies were not checked to evaluate for VITT, the patient tested negative for COVID-19 infection, and he had no other predisposing risk factors for developing acute venous thrombosis. He was generally active, working in the construction industry and using the gym three times a week; therefore, the development of lone DVT and pulmonary embolism due to the mRNA-1273 vaccine is the most plausible explanation. Also causes of clots other than VITT, with a predilection for the venous circuit after receiving mRNA vaccines were reported before. Researchers reported cases of venous thrombosis following COVID-19 vaccine administration with normal platelet counts and negative PF4 antibodies supporting our theory that VITT is not the only reason for VTE following vaccination against COVID-19 [12,13].

Scientists have discussed the similarity between COVID-19 virus and COVID-19 vaccines [14]. As the virus has a high thrombogenic effect, the same thrombogenicity can happen after COVID-19 vaccination. Although the mechanism behind this is unclear, observers noticed the association between elevated factor V activity and VTE [15]. Additionally, the spike proteins generated by mRNA vaccines can create a cascade of events leading to endothelial dysfunction and subsequently the development of venous thrombosis [13].

Multiple well-known risk factors can lead to VTE. Examples are recent trauma to the leg, recent surgeries, family history of thrombosis, and prolonged immobility. Our patient did not have any of these risk factors. About 40% of the DVT in the general population can be idiopathic in origin [16]. The patient did not follow up in the outpatient settings for a complete thrombophilia workup and to rule out the possibility of an underlying uncommon clotting disorder. The lack of PF4 antibodies testing does not rule this case out, as both VITT and non-VITT causes have been associated with the development of thromboembolic disease following vaccination against COVID-19 infection. One study assessed the association between COVID-19 vaccines and adverse hematological outcomes. Both mRNA and viral vector vaccines against COVID-19 infection increase the risk of hospital admissions because of hematological side effects. Still the incidence of these unfavorable outcomes after COVID-19 vaccination are much lower as compared to after COVID-19 infection [17].

## Conclusions

Although some reports suggest an association between COVID-19 vaccines with the development of VTE, we still recommend COVID-19 vaccination. The risk of developing VTE from the vaccine is significantly lower than the VTE correlated to the actual COVID-19 infection; the hypercoagulable effects of COVID-19 are well established in the literature. Future studies and robust database monitoring can help elicit the true incidence of COVID-19 vaccine-induced thrombosis as more adverse reporting data is collected.
